# Crosstalk Between Auditory and Visual information in Motor Learning and Savings

**DOI:** 10.1111/ejn.70502

**Published:** 2026-04-12

**Authors:** Olivier White

**Affiliations:** ^1^ INSERM UMR1093‐CAPS, Université Bourgogne Franche‐Comté, UFR des Sciences du Sport Dijon France

**Keywords:** action and decision making, internal models, motor adaptation, perception, savings, skill transfer

## Abstract

Sensorimotor adaptation relies on the integration of multisensory feedback, yet the specific contributions of auditory signals to this process remain poorly understood compared to vision. We tested 46 participants on a reaching task using visual, auditory, or combined feedback about terminal errors. Participants alternated between feedback modalities during learning and relearning, revealing how adaptation transfers across sensory systems. Strikingly, although auditory feedback alone failed to support initial learning, prior visual learning enabled subsequent auditory adaptation. Further experiments showed that this cross‐modal transfer stems from a memory of the learned reaching direction, acting as an attractor for movement, independent of error‐based mechanisms, or explicit strategies. Our findings unveil a novel pathway for sensory integration in motor learning, with implications for designing cross‐modal training protocols.

## Graphical abstract

Visual feedback enables motor adaptation, whereas auditory feedback alone fails to support initial learning. Crucially, prior visual learning allows for subsequent auditory adaptation. This cross‐modal transfer stems from a memory of the successful reaching direction, which acts as a movement attractor independent of sensory modality. These results suggest that savings are driven by reward‐based directional memories rather than error‐based recalibration.

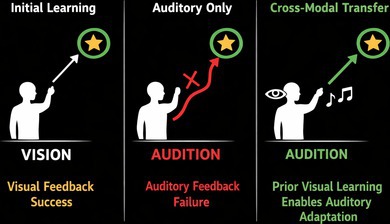

**Abbreviations**
A → V: auditory‐to‐visual feedback group
V → A: visual‐to‐auditory feedback group
‐ V → A: visual‐to‐auditory group with reversed rotation
V + A: combined visual and auditory feedback group

## 1 Introduction

The brain relies on sensory feedback to correct inaccurate movements and improve the outcome of subsequent actions. The body is equipped with different biological sensors that serve this purpose. For instance, vision (Held and Freedman 1963) and proprioception (Wolpert et al. 1995; Sarlegna and Sainburg 2009) are known to yield critical and complementary information (van Beers et al. 2002). Surprisingly, little attention has been paid to audition (Opoku‐Baah et al. 2021) despite the fact this inflow is the main sensory feedback modality for speech movements. Humans modulate the loudness of their speech in function of the loudness of the environment and as a function of how they perceive their own voice (Lane and Tranel 1971). Similarly, artificially altering the auditory feedback results in a modification of the vocal output (Chen et al. 2007). If such modifications are maintained consistently for several exposures, then it leads to a long‐term change in speech production (Houde and Jordan 1998). Finally, a study also showed that auditory feedback can enhance movement timing (van Vugt and Tillmann 2015).

Both visual and auditory feedback modalities can be used to guide upper limb and speech movements. For instance, visual information about one's own speech movements can improve language training (Katz and Mehta 2015). Similarly, the ability to use auditory feedback to maintain optimal motor output extends beyond speech production. When sound is not consistent with an action (e.g., delayed auditory feedback), it may interfere with the smooth course of an action and even result in destructive interactions. Altering auditory feedback during the performance of sequential motor actions such as playing the piano interferes with the production of the melody (Pfordresher 2005, 2012; Furuya and Soechting 2010). Audio‐motor interactions are numerous while playing musical instruments (Zatorre et al. 2007; Cao et al. 2016) or, more generally, in other types of tasks (Sigrist et al. 2013). Proper use of auditory information can also result in constructive interaction. For instance, providing auditory feedback about motor performance is sufficient to induce reliable learning in a natural gait learning task (Zanotto et al. 2013) or in a trunk‐arm rowing task (Sigrist et al. 2014) and also in a classical experimental cursor motion tracking paradigm (Rosati et al. 2012). Auditory feedback can be used for sensorimotor adaptation of reaching movements even though such adaptation mainly relies on visual and proprioceptive information. Changing the location and/or loudness of the sound allows humans to derive the motor error and to adapt to a perturbation (Oscari et al. 2012; Schmitz and Bock 2014). This suggests that sensory substitution of visual information by auditory information is sufficient to induce motor adaptation. But are auditory and visual information treated equally by the brain or do they provide complementary information?

Our main interest was to study how learning with one modality (e.g., visual) could be transferred to relearning in another modality. Savings refers to faster relearning upon re‐exposure to a previously encountered perturbation, operationally defined as a larger reduction in error during the initial trials of relearning compared to initial learning. This well‐known phenomenon has been observed in a wide range of declarative (e.g., Ebbinghaus 2013) and motor learning tasks (Brashers‐Krug et al. 1996; Krakauer et al. 2005). In motor adaptation tasks, several mechanisms have been proposed to account for savings. Some authors suggested that savings could arise from a memory of errors (Herzfeld et al. 2014), from the reliance on previous motor memories (Smith et al. 2006), from the use of an explicit strategy (Haith et al. 2015; Morehead et al. 2015), and/or from a reward‐based memory of successful motor actions (Huang et al. 2011; Shmuelof et al. 2012; Orban de Xivry and Lefèvre 2015) as well. Although recent work has advanced our understanding of these mechanisms—for instance, Hadjiosif et al. (2023) demonstrated a double dissociation between savings and long‐term memory—the relative contributions of error‐, strategy‐, and reward‐based processes remain debated.

Whereas experimental manipulations exist to minimize the contribution of the explicit strategy (Taylor et al. 2014; Morehead et al. 2015) or the reliance on previous motor memories (Huang et al. 2011; Orban de Xivry and Lefèvre 2015), reducing the contribution of memory of errors mechanisms has proven more difficult. Here, we reasoned that if savings were observed across modalities, it could not be due to a memory of errors given their different modalities. Internal model theory predicts that adaptation should be modality specific, as the sensory prediction errors driving implicit recalibration depend on the feedback modality. In contrast, reward‐based memory theory predicts that savings could transfer across modalities, because the memory of successful motor actions is encoded in terms of the movement direction rather than the sensory feedback that signaled success. In a first experiment, we tested the transfer of motor adaptation between visual and auditory sensory modalities. In a second experiment, we tested whether the observed savings are due to an explicit strategy or to a memory of the successful motor actions learned during the first exposure (Huang et al. 2011; Orban de Xivry and Lefèvre 2015).

## 2 Materials and Methods

### 2.1 Participants

Forty‐six right‐handed adults were included in this study. Experiment 1 comprised 34 participants (17 females, 24.3 ± 3.9 years old), and Experiment 2 involved 12 different participants (8 females, 23 ± 2.2 years old). All participants were volunteers, healthy, without neuromuscular disease or auditory impairment, and with normal or corrected to normal vision. Furthermore, participants were musically trained. Musical training was defined as having received at least 3 years of formal musical instruction or regular practice on a musical instrument, assessed via self‐report questionnaire and used as an inclusion criterion. Participants reported an average of 6.2 ± 2.5 years of musical practice. The experimental protocol was carried out in accordance with the Declaration of Helsinki (1964), and the procedures were approved by the ‘Comité d'Ethique pour les Recherches’ (CER) of Université de Bourgogne. All participants were naïve as to the purpose of the experiments and were debriefed after the experimental session.

### 2.2 Apparatus and Stimuli

Participants were comfortably seated in front of a virtual environment equipment with the head on a chin rest in a quiet, dimly illuminated room. They looked into two mirrors that were mounted at 90° to each other, such that they viewed one LCD screen with the right eye and one LCD screen with the left eye. This stereo display was calibrated such that the physical location of the robotic arm was consistent with visual disparity information. Participants made 12‐cm movements while holding on to a robotic device with the right hand (Phantom 3.0, SensAble Technologies, United States). Movements were performed in the natural reaching space in an upward‐forward direction, involving shoulder and elbow movements, with the elbow pointing downward. The experiments took less than 75 min, including briefing and debriefing.

### 2.3 Experimental Procedure

**FIGURE 1:**
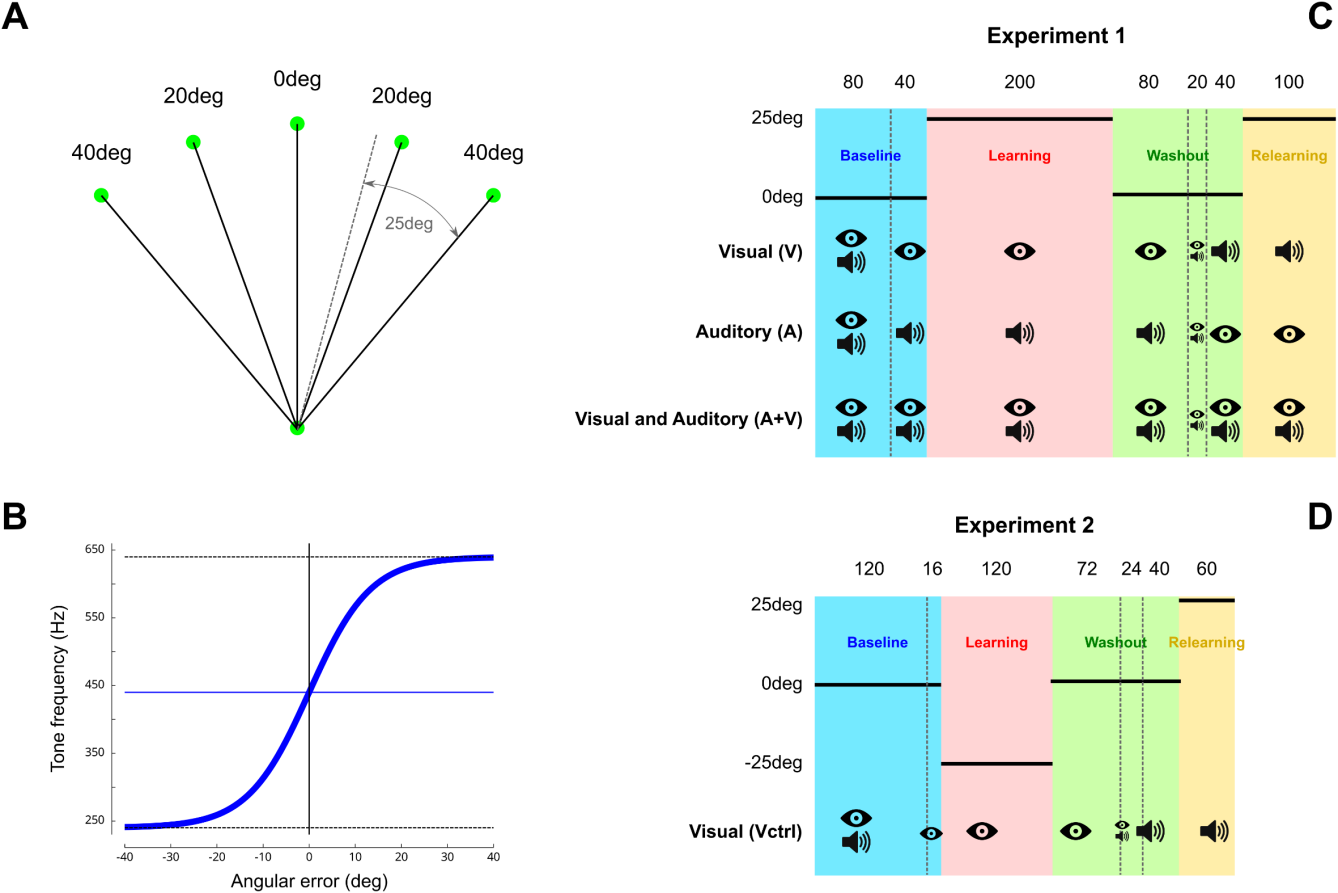
Illustration of experimental procedures. (A) Scaled trajectories of reaching movements toward the five targets in Experiment 1 (top circles). The dotted line represents a successful shooting movement toward the 40° target after full adaptation to a +25° rotation in hand space. (B) Pitch of the feedback sound (*y* axis) in function of the angular error (*x* axis). Resolution is optimized around 0 error (largest slope). The two horizontal dashed lines represent the saturation pitches (240 and 640 Hz), and the horizontal solid line is positioned at the reference frequency of 440 Hz. The vertical cursor is positioned at the angular error of 0°. (C) In Experiment 1, each group (rows) received different feedback modalities but was exposed to the same perturbation schedule. An “eye” icon and a “speaker” icon correspond to the presence of visual (V), auditory (A), or both (V + A) feedback information. The numbers on top represent the number of trials for each period, separated by dashed vertical and by gray rectangles. (D) Experimental schedule for Experiment 2.

Participants performed shooting movement toward one of five green targets positioned on a circle (radius = 12 cm) at five different eccentricities (−40°, −20°, 0°, 20°, and 40°). The 0° direction corresponded to the vertical upward direction (Figure 1A). The 3D positions of the robot handle were mapped in real time to a gray cursor (diameter = 3 mm). A trial started when the cursor was positioned inside a starting green sphere (diameter = 6 mm). Then, as soon as one of the five targets appeared (diameter = 6 mm), the participant performed a rapid shooting movement through the target. Continuous visual feedback of the cursor trajectory was not provided. The trial ended when the radial distance from the starting position and the cursor was larger than 12 cm. We instructed and trained participants to reach the target within around 250 ms. After each trial, the target turned red or blue if movement times were longer than 300 ms or shorter than 200 ms, respectively.

After 500 ms, feedback about shooting error was provided through two different modalities. In the visual modality, a yellow cursor (diameter = 6 mm) was flashed during 1 s at the position closest to the target. The error was therefore signed; the yellow cursor could appear on the left or on the right of the target. In the auditory modality, error information was provided through a sound delivered through headphones at a comfortable volume (JBL) defined the following way. A continuous sound was emitted for 1 s but with specific frequencies. During the first 500 ms, its pitch followed a sigmoid function defined by

$$f\left(\alpha \right)=f_{0}\left(1-\frac{1}{1+\exp\left(-\tau \alpha \right)}\right)+f_{1}\frac{1}{1+\exp\left(-\tau \alpha \right)}.$$

Pitch saturated at frequency *f*_0_ or *f*_1_ for angular errors α larger than −30° or +30°, respectively (Figure 1B). The inflection point and largest slope of the sigmoid function were set at 0° error in order to maximize the resolution of the auditory feedback around target position. The parameters were set as follows: *f*_0_ = 240 Hz, *f*_1_ = 640 Hz, and *τ* = 0.15°^−1^. During the 500 ms that followed, a single frequency tone of 440 Hz that corresponded to a “A4” in the main octave was played back. This tone provided a reference sound for a perfect shooting movement (error α = 0°). Participants interpreted the sign of the error by comparing the trial and reference pitches, these aptitudes being well developed in humans (Plack and Oxenham 2005). Trial frequencies lower (upper) than 440 Hz were interpreted as angular errors to the left (right) of the target, and no error resulted in no identified differences between pitches. The 500‐ms sound modulated by the angular error was emitted immediately after the trial in order to reinforce the link between the movement just performed and its auditory consequences. After the trial ended, the robot moved the hand back onto the starting circle. This sped up trials and prevented participants performing active return movements.

In Experiment 1, participants were randomly dispatched in one of three groups, depending on feedback modality. In the “VISUAL” group (V → A, *n* = 12), endpoint error was provided through visual modality and then through auditory modality. It was the opposite in the “AUDITORY” group (A → V, *n* = 11). A third group was provided with both modalities for all trials (“AUDITORY and VISUAL,” V + A, *n* = 11).

After briefing, participants were familiarized with a practice block of 20 trials to a single target at −15° that were not considered for analysis. Cursor trajectories were not displayed online, and feedback was provided both in the visual and auditory modalities. During the experimental session, participants performed 560 trials in the following order (Figure 1C). The baseline block consisted of 120 reaching movements (24 to each target) to randomly selected targets (Figure 1C, “baseline” sequence). During the first 80 trials, feedback was visual and auditory, whereas it was either visual, auditory or both, depending on the group, for the last 40 trials. Then, a visuomotor perturbation was introduced for the next 200 shooting movements (40 trials × 5 targets). The perturbation consisted of a +25° clockwise rotation of the cursor for all groups. However, groups relied on different feedback to adapt their shooting movements (Figure 1C, “learning” sequence). During the perturbed trials, participants had to move the hand 25° to the left of the target to perform a successful trial (Figure 1A, dotted lines). After this perturbation block, 140 trials were again performed in the same condition as baseline (Figure 1C, “washout” sequence), without visuomotor rotation. During the first 80 trials, only feedback modality of that group was provided, like in the previous sequence. Next, participants could use both feedback modalities to adjust their movement in a short block of 20 trials. Finally, during the last 40 trials of this “washout” sequence, participants switched to the other feedback modality (i.e., “auditory” for the “VISUAL” group and vice versa). The “VISUAL and AUDITORY” group had always received both feedback modalities. In the last sequence (Figure 1C, “relearning”), the same perturbation was reintroduced in the other modality for 100 trials.

In Experiment 2 (*n* = 12), we specifically tested for the contribution of an explicit strategy or of a reward‐based memory of the learned movement direction. The procedure was similar to the *VISUAL* group in Experiment 1 and differed only for target positions and number of presentations (Figure 1D). The baseline block consisted of 120 reaching movements to one of four targets (−90°, −40°, 10°, or 60°) based on Figure 1C (“baseline” sequence). In the “learning” sequence, only three targets were presented (−90°, −40°, and 10°), and hand cursors were rotated 25° counterclockwise (−25°). To avoid overlearning, block design ensured that the same number of trials per target was presented in this condition (40 trials per target × 3 targets = 120 trials). The “washout” sequence consisted in 136 shooting movements to the same four targets as in the baseline sequence without visuomotor rotation. Finally, relearning was tested in the “auditory” modality but with different targets (−40°, 10°, and 60°) and with the opposite perturbation (clockwise, 25°). The new set of targets was arranged so that the same hand trajectories (−65°, −25°, and 15°) as during the learning led to success. In other words, this experimental design allowed us to test savings. In hand space, the solution to the second perturbation of three targets ([−40°, 10°, 60°]−25° = [−65°, −15°, 35°]; “relearning”) was identical to the solution of the first perturbation ([−90°, −40°, 10°] + 25° = [−65°, −15°, 35°], “learning”).

### 2.4 Data Processing

Positions were recorded with a sampling rate of 500 Hz. Movement start was detected when movement velocity exceeded 3 cm/s for at least 100 ms. Angular error of each movement was defined as the angular deviation at the end of the movement from the straight direction toward the target. In Experiment 1, we quantified, for each participant, the amount of learning, washout, and relearning by looking at the difference between the mean of the angular errors during the first five trials (early) and the mean of the angular errors during the last 15 trials (late) in each sequence. We adopted the same procedure for Experiment 2 to account for the different number of targets among the three sequences. Namely, learning and relearning (using three targets) were assessed using the first 3 and last 9 trials, whereas washout (using four targets) was quantified by comparing the difference in angular errors between the first 4 trials and the last 12 trials in that sequence.

The existence of savings was assessed by quantifying the speed of learning. To do so, we fitted an exponential function of the form $a_{1}\exp\left(-a_{2}t\right)+a_{3}$ to the angular error, where *a*_1_, *a*_2_, and *a*_3_ are constants quantifying the amplitude of adaptation, the adaptation rate, and the asymptote, respectively.

To compare learning and savings across groups, we adopted mixed‐design ANOVAs with group (V → A, A → V, V + A) as the between‐subjects factor and time (early vs. late adaptation) as the within‐subjects factor. One‐way ANOVAs were used to compare differences between groups at specific experimental phases. When ANOVAs yielded significant main effects or interactions, Tukey HSD post hoc tests were performed to identify specific pairwise differences. For within‐group comparisons of specific conditions, paired *t* tests were used, whereas independent *t* tests were applied for specific between‐group contrasts. Quantile‐quantile (*Q*‐*Q*) plots were used to assess the normality of the data. Effect sizes are reported as partial eta‐squared for ANOVAs and Cohen's d for *t* tests. Sample size was fixed at 10+ subjects per group before the start of the experiments, a number chosen to provide sufficient power for between‐subject effects. Data processing and statistical analyses were performed using Matlab (The Mathworks, Natick, MA).

## 3 Results

In this experiment, we test the effect of visual versus auditory feedback on the adaptation to a visuomotor rotation and on the transfer of learning between these two modalities. We reasoned that such transfer would highlight the role of nonerror‐based mechanisms in savings because error‐based saving mechanisms should depend on the modality of the error feedback. Participants from the V → A group received visual feedback during learning and auditory feedback during relearning. The second group (A → V) received auditory feedback during learning and visual feedback during relearning. Finally, we expected maximal adaptation for the participants who received both visual and auditory feedback during the entire experiment (V + A group). Except for the modality of the error feedback, the participants from these three groups were exposed to an identical schedule of visuomotor perturbations (Figure 1C).

### 3.1 Inability to Adapt Under Auditory Error Feedback

**FIGURE 2:**
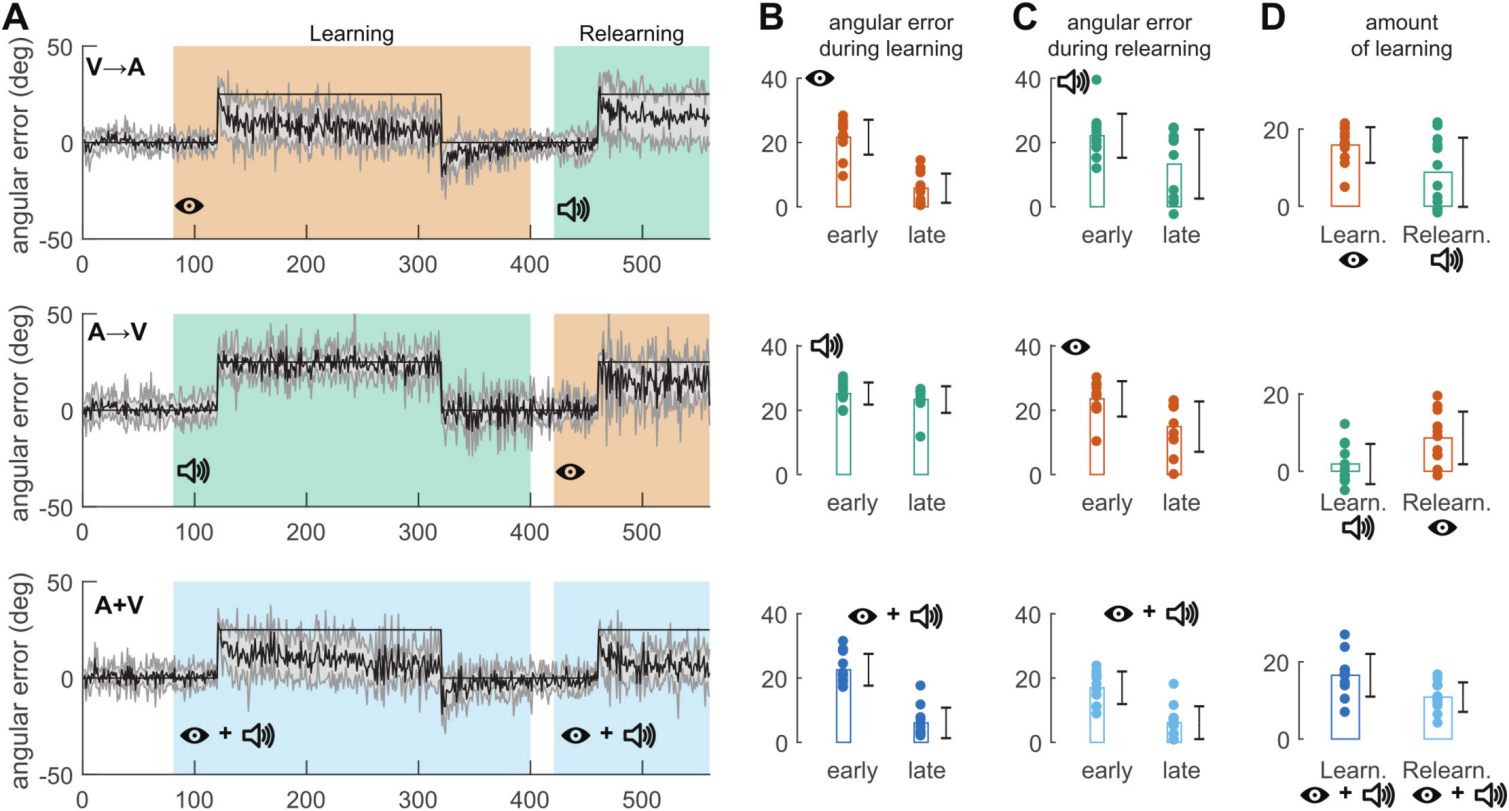
Evolution of the angular error over trials for the three groups of Exp. 1 (first row: V → A; second row: A → V; third row: V + A). (A) Evolution of the angular error over the course of the experiment. Data are averaged across participants (solid line), and gray shaded area correspond to the standard error of the mean. Thin black line corresponds to cursor rotation. Green background: auditory feedback. Orange background: visual feedback. Blue background: auditory and visual feedback. (B) Average angular error early and late during the learning period. Early = first cycle (five trials); late = last six cycles (30 trials). (C) Average angular error early and late during relearning. (D) Average amount of learning during the learning (early) and relearning (late) periods. For (B)–(D), color code is similar to (A). On each plot, individual values are represented by dots for each measure. Rectangles represent the mean. Black error bars represent mean ± SE.

After baseline trials, each group was confronted with a 25° visuomotor rotation (Figure 2). Initially, the perturbation caused identical initial angular errors in the three groups (main effect of group on initial angular error: *F*_2, 31_ = 1.74, *p* = 0.19, $\eta_{p}^{2}=0.1$). However, adaptation to the perturbation was absent in the group learning under auditory feedback (A → V), whereas the two other groups (V → A and V + A) could adapt normally to the perturbation (learning period in Figure 2A). The absence of learning under auditory feedback about terminal errors resulted in a larger final error for the A → V group compared to the two other groups (Figure 2, second column), yielding a significant effect of group on the final error measure (*F*_2, 31_ = 56.25, *p* < 10^−10^, $\eta_{p}^{2}=0.78$). A Tukey post hoc test to compare the final error across groups revealed that the group that learned in the “auditory” modality (A → V) was different from the two other groups (A → V vs. V → A: *p* < 10^−8^ and A → V vs. V + A: *p* < 10^−8^). In other words, participants from the A → V group did not decrease their angular error as much as the two other groups (interaction between group and time, initial vs. late error: *F*_1, 31_ = 58.17, *p* < 10^−7^, $\eta_{p}^{2}=0.52$). Indeed, late angular errors were significantly smaller than initial errors in the V → A group (*t* test: *t*_11_ = 11.9, *p* < 10^−6^, *d* = 3.44) and V + A group (*t*_10_ = 9.31, *p* < 10^−5^, *d* = 2.81) but not in the A → V group (*t*_10_ = 1.05, *p* = 0.318) that received auditory feedback only during the learning period.

The lack of adaptation of the A → V group was confirmed by the limited after‐effect for this group in the ensuing washout period under the same feedback modality (mean ± SD: −2.5° ± 3.8°), whereas the two other groups demonstrated a much larger after‐effect (V → A: −13.57° ± 3.6° and V + A: −11.4° ± 5.9°). The after‐effect of the A → V group was significantly smaller than in the other two groups (main effect of group: *F*_2, 31_ = 19.03, *p* < 10^−5^, $\eta_{p}^{2}=0.55$; post hoc Tukey test: A → V vs. V → A: *p* < 10^−5^; A → V vs. A + V: *p* = 0.0002; and V → A vs. V + A: *p* = 0.49).

Participants from all groups then performed 20 trials with concurrent visual and auditory feedback without perturbation (Figure 1C), which were followed by 40 unperturbed shooting movements but with only the other feedback modality. In other words, the V → A and A → V groups switched to the other feedback modality (the V + A group still received V + A feedback). By the end of this washout phase, all groups performed reaching movements without significant errors (V → A: *t*_11_ = 1.13, *p* = 0.28; A → V: *t*_10_ = 0.76, *p* = 0.463; V + A: *t*_10_ = 0.57, *p* = 0.583).

### 3.2 Relearning Under Auditory Feedback Is Possible After Learning Under Visual Feedback

In the relearning period, participants from the V → A and A → V groups were exposed to the same perturbation a second time but under the other modality. That is, participants who experienced the first perturbation under auditory (visual) feedback received visual (auditory) feedback during the relearning period. Participants of the V + A group received both modalities during the relearning period as well.

Surprisingly, in this second learning period, adaptation to perturbation was observed under auditory feedback. Indeed, participants from the V → A group changed their reaching direction during the relearning period (change in angular error from start to end of relearning period: *t*_11_ = 3.4, *p* = 0.006, *d* = 0.98) even though they only received auditory feedback during that period. For this group, the difference between the learning under visual feedback and the relearning under auditory feedback did not reach significance (interaction between period of learning and time (early vs. late): *F*_1, 11_ = 4.62, *p* = 0.05, $\eta_{p}^{2}=0.3$). More importantly, this change in error was larger than the change observed during the first 100 trials of the learning period under auditory feedback in the A → V group (learning A → V group vs. relearning of the V → A group, interaction between group and time: *F*_1, 21_ = 7.88, *p* = 0.01, $\eta_{p}^{2}=0.27$).

The A → V group that received the auditory feedback during the learning period—and exhibited no learning—was well able to learn the visuomotor rotation under visual feedback (difference between initial and final errors in the relearning period: *t*_10_ = 4.2, *p* = 0.002, *d* = 1.27). This relearning was significantly larger than the change observed in the learning period of this group, under auditory feedback (interaction between period and time: *F*_1, 10_ = 23.7, *p* = 0.0006, $\eta_{p}^{2}=0.7$). This suggests that this group was able to adapt to a visuomotor rotation under normal visual feedback condition but that the auditory feedback as provided here is insufficient to drive motor adaptation. Overall, we could not find any difference in the amount of relearning across the three groups (ANOVA on angular error, interaction between group and time: *F*_2, 31_ = 0.35, *p* = 0.7, $\eta_{p}^{2}=0.02$).

Exponential fitting (mean *R* ^2^ = 0.21) corroborated these findings. The adaptation rate (*a*_2_) was higher in the V → A group during auditory relearning (0.151 ± 0.062 SEM) compared to the A → V group during initial auditory learning (0.033 ± 0.015; *t*_11_ = 1.86, *p* = 0.089), where fits were often poor or nonconvergent. In contrast, visual adaptation rates remained consistent regardless of prior auditory exposure (V → A, initial *a*_2_ = 0.123 vs. A → V relearning *a*_2_ = 0.126), confirming that savings were specific to the V → A transition.

### 3.3 Relearning Under Auditory Feedback Is Not due to an Explicit Strategy but to a Memory of the Learned Directions

**FIGURE 3:**
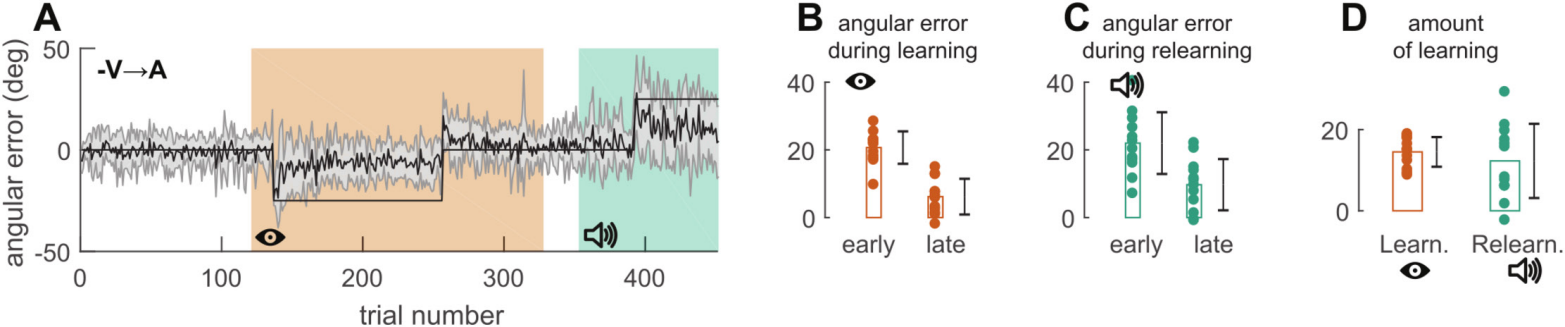
Evolution of the angular error over trials for Exp. 2. (A) Evolution of the angular error over the course of the experiment. Data are averaged across participants (solid line) and gray shaded area correspond to the standard error of the mean. Thin black line corresponds to cursor rotation. Green background: auditory feedback. Red background: visual feedback. (B) Average angular error early and late during the learning period. Early = first cycle (three trials); late = last six cycles (18 trials). (C) Average angular error early and late during relearning. For (B)–(D), color code is similar to (A). On each plot, individual values are represented by dots for each measure. Rectangles represent the mean. Black error bars represent mean ± SE.

Two possible components of motor adaptation could account for the observed relearning under auditory feedback in the V → A group. Either participants adopted an explicit strategy and modified their reach angle consciously in the same direction as during the learning period, or they implicitly converged toward the learned reaching direction that was rewarded at the end of the learning period. To differentiate between these two hypotheses, we conducted an additional control experiment (Experiment 2, Figure 3) directly inspired by Experiment 3 of Huang et al. (2011). In our experimental design, rotation angles between the learning (−25°) and relearning periods (+25°) are reversed. Therefore, the use of an explicit strategy would lead to an increase in error early during the relearning period. In contrast, the targets are arranged in such a way that the hand directions that counteract the visuomotor rotation are identical during the two adaptation periods. Therefore, the hand direction learned during the first adaptation period could drive the learning in the second adaptation period through reward‐based learning even though the change in hand direction is opposite in these two periods.

Figure 3 presents results from Experiment 2. Participants from this group (−V → A) adapted first to a −25° visuomotor rotation under visual feedback (difference between early and late angular error, *t*_11_ = 13.74, *p* < 10^−7^, *d* = 3.97), which yielded a significant after‐effect (mean ± SD: 12.2 ± 6.2; *t*_11_ = 6.8, *p* < 10^−4^, *d* = 1.96). Most importantly, we observed a decrease in error during the relearning period (mean ± SD: 12.3 ± 9.1; *t*_11_ = 4.66, *p* = 0.0007, *d* = 1.35), consistent with the hypothesis that the reward‐based memory of hand direction was able to guide the learning during the second adaptation period and that the explicit strategy was not responsible for the observed learning under auditory feedback during the second adaptation period.

**FIGURE 4:**
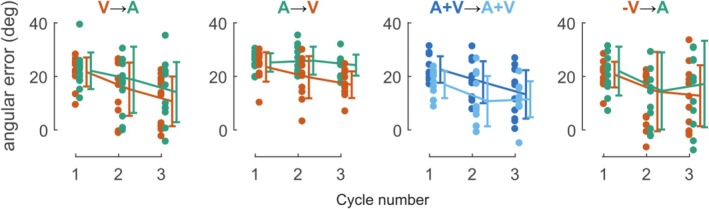
Evolution of the angular error during the first three cycles of the learning and relearning periods for the four experimental groups. Orange, green, and blue correspond to visual (V), auditory (A), or both modalities (A + V) during learning (before the arrow) or relearning (after the arrow). Each cycle corresponds to one presentation of each target.

The use of explicit strategy in the control experiment could be seen in an increase in the angular error during the first cycles of the relearning periods. However, such a change in strategy was not apparent in our data as shown in Figure 4.

## 4 Discussion

In the present study, we demonstrated that substituting visual error information with auditory error information did not allow the participants to adapt to a 25° visuomotor rotation. However, if adaptation to the perturbation by means of auditory error information was preceded by a first exposure to the same perturbation combined with visual error information, then participants were able to adapt to the perturbation based on auditory error information. Our control experiment suggests that adaptation under auditory feedback in the relearning period is driven by a reward‐based memory of the rewarded hand direction and not by an explicit strategy. Together, these results suggest that transfer of adaptation under different sensory feedback modalities is possible through reward‐based motor memory that can act as an attractor for the reaching direction.

### 4.1 Absence of Learning Based on Auditory Error Information on the First Exposure

Humans are particularly skilled at detecting differences in pitch frequency (Bendor and Wang 2006). All participants played at least one musical instrument. However, on average, adaptation did not emerge when participants received auditory feedback during the first exposure. This finding is consistent with a previous study that showed that aftereffects were absent in auditory retention trials, whereas aftereffects remained high during visual retention trials (Kagerer and Contreras‐Vidal 2009). However, this also contrasts with previous studies (Oscari et al. 2012; Zanotto et al. 2013; Schmitz and Bock 2014). These differences might be due to the experimental setup and to the way feedback was provided. First, we provided endpoint auditory feedback, whereas previous studies used online auditory feedback. It is known that online and endpoint feedback influence motor adaptation in different ways (Taylor et al. 2013). Second, we used the content of the auditory stimulus rather than its localization to provide feedback (Schmitz and Bock 2014). This forces the participants to build an arbitrary mapping between the content of the auditory stimulus and the error they performed. This new relationship is very common when we cope with visuomotor rotations. For instance, moving a computer mouse on a horizontal table induces cursor displacements in the vertical plane (screen) with a gain usually different from 1. Third, there was a short delay between the end of the movement and the presentation of the auditory feedback, which might hamper the adaptation process (Brudner et al. 2016). This delay was the same in the VISUAL and AUDITORY modalities. Because the 500‐ms delay was identical across modalities and the trial design was self‐paced, timing alone cannot explain the initial auditory deficit, as participants had sufficient time to process feedback regardless of sensory network. Furthermore, our exponential fitting analysis confirms that the abstract mapping is not inherently “unlearnable” but becomes accessible to the motor system only once a spatial reference frame has been established through prior visual exposure.

The absence of adaptation when auditory feedback alone was provided suggests that implicit adaptation of the internal model (Mazzoni and Krakauer 2006; Morehead et al. 2017; Kim et al. 2018) might not take place under auditory feedback alone, as suggested by another study (Kagerer and Contreras‐Vidal 2009).

### 4.2 Reward‐Based Memory Acts as an Attractor

Interestingly, we found that, under auditory feedback, participants adapted to the perturbation during the second exposure but not during the first exposure. We believe that the learning on the basis of auditory feedback during the second exposure is driven by the spontaneous recovery of the motor memory (here a movement direction) elicited by the absence of reward (Pekny et al. 2011), which acts as an attractor for the hand movement (Shmuelof et al. 2012). Our findings suggest that the motor memory formed during initial visual learning does not simply fade but persists as a latent spatial attractor—a stored representation of the rewarded hand direction that biases subsequent motor commands toward the previously successful solution, even when the sensory feedback context changes entirely. Although internal models may facilitate the fine‐tuning of movements within a modality, the robust cross‐modal savings observed here suggest that the “memory of success” (reward based) is the primary driver of rapid readaptation when the sensory environment changes. It is unsure whether the content of the auditory feedback played any role in the observed phenomenon. Indeed, given that the A → V group demonstrated that participants were unable to use the auditory feedback to adjust their hand movement to the visuomotor rotation, the observed effect might be driven by the removal of the reward at the end of each movement. In our experiment, successful movements were rewarded by points. Removing this rewarding signal yielded to spontaneous recovery of the motor memory as observed in our study and in others (Pekny et al. 2011; Shmuelof et al. 2012). This is also consistent with the savings observed in Experiment 3 of Huang et al. (2011) and in Orban de Xivry and Lefèvre (2015). Indeed, our control experiment that adopted a design similar to Huang et al. shows that the sign of the error during the first exposure did not matter and that the learning during the second exposure was due to the recall of the reward‐based memory, which acted as an attractor for the hand movement. The learning in the second exposure through auditory feedback cannot be due to a memory of errors (Herzfeld et al. 2014), as the errors are radically different between the visual and auditory feedback modalities. Crucially, Experiment 2 demonstrates that the mere experience of errors is insufficient to trigger savings if those errors do not lead to a successful outcome. This further reinforces the reward‐based account: Savings in our task were not a result of ‘learning to learn’ through error repetition but were specifically tied to the reinforcement of the successful 30° compensated trajectory. It is also unlikely that it is due to an explicit strategy as the explicit strategy would have driven the hand in the opposite direction in the control experiment (Morehead et al. 2015), which was not observed in the present study.

### 4.3 Limitation

Several limitations should be acknowledged. First, the auditory feedback used a frequency‐based, nonspatial mapping. Although this was a deliberate choice to probe abstract auditory error coding, it remains possible that more ecologically valid auditory feedback—such as spatially lateralized sounds—could support robust initial adaptation and alter the pattern of cross‐modal savings observed here. Second, although all participants met a minimum criterion of musical training (≥ 3 years), no formal psychophysical pitch‐discrimination task was administered. Including such a measure would have provided direct confirmation that participants could reliably interpret the feedback signal. Third, the musically trained sample may limit generalizability to untrained populations. Fourth, the single‐session design precludes conclusions about the long‐term retention of cross‐modal savings. Future studies should examine whether the reward‐based motor memory identified here persists over days or weeks and whether it generalizes to other types of perturbations and feedback mappings.

### 4.4 Conclusion

We tested the independence of a reward‐based motor memory of movement direction on the sensory modality during which it was created and found that reward‐based memory can be formed and recalled independently of the sensory modality. This reward‐based memory allowed the participants to learn a task that they could not learn otherwise independently of any memory of errors or explicit strategy. We believe that this study presents a new way of testing the presence and strength of a reward‐based motor memory. In conclusion, the crosstalk between auditory and visual information in our task suggests that the brain builds a higher order representation of motor success. This suggests that motor memories are more abstract and flexible than previously thought, allowing for the seamless transfer of learned skills across different sensory contexts.

## Author Contributions

**Olivier White:** conceptualization, data curation, formal analysis, funding acquisition, investigation, methodology, project administration, resources, software, supervision, validation, visualization, writing – original draft, writing – review and editing.

## Funding

This work was supported by the Fonds Européen de Développement Régional (FEDER), the Conseil Général de Bourgogne, and the Institut National de la Santé et de la Recherche Médicale.

## Ethics Statement

The experimental protocol was carried out in accordance with the Declaration of Helsinki (1964) and the procedures were approved by the “Comité d'Ethique pour les Recherches” (CER) of Université de Bourgogne.

## Conflicts of Interest

The author declares no conflicts of interest.

## Data Availability

The data that support the findings of this study are available on request from the corresponding author.

## References

[ejn70502-bib-0001] Bendor, D. , and X. Wang . 2006. “Cortical Representations of Pitch in Monkeys and Humans.” Current Opinion in Neurobiology 16: 391–399. 16842992 10.1016/j.conb.2006.07.001 PMC4325365

[ejn70502-bib-0003] Brashers‐Krug, T. , R. Shadmehr , and E. Bizzi . 1996. “Consolidation in Human Motor Memory.” Nature 382 252–255. 8717039 10.1038/382252a0

[ejn70502-bib-0004] Brudner, S. N. , N. Kethidi , D. Graeupner , R. B. Ivry , and J. A. Taylor . 2016. “Delayed Feedback During Sensorimotor Learning Selectively Disrupts Adaptation but Not Strategy Use.” Journal of Neurophysiology 115: 1499–1511. 26792878 10.1152/jn.00066.2015 PMC4808111

[ejn70502-bib-0005] Cao, Y. , B. L. Giordano , F. Avanzini , and S. McAdams . 2016. “The Dominance of Haptics Over Audition in Controlling Wrist Velocity During Striking Movements.” Experimental Brain Research 234: 1145–1158. 26790425 10.1007/s00221-015-4529-9 PMC4785215

[ejn70502-bib-0006] Chen, S. H. , H. Liu , Y. Xu , and C. R. Larson . 2007. “Voice F0 Responses to Pitch‐Shifted Voice Feedback During English Speech.” Journal of the Acoustical Society of America 121: 1157–1163. 17348536 10.1121/1.2404624

[ejn70502-bib-0007] Ebbinghaus, H. 2013. “Memory: A Contribution to Experimental Psychology.” Annals of Neurosciences 20, no. 4: 155–156. 25206041 10.5214/ans.0972.7531.200408 PMC4117135

[ejn70502-bib-0008] Furuya, S. , and J. F. Soechting . 2010. “Role of Auditory Feedback in the Control of Successive Keystrokes During Piano Playing.” Experimental Brain Research 204: 223–237. 20521031 10.1007/s00221-010-2307-2 PMC3179864

[ejn70502-bib-0009] Hadjiosif, A. M. , J. R. Morehead , and M. A. Smith . 2023. “A Double Dissociation Between Savings and Long‐Term Memory in Motor Learning.” PLoS Biology 21, no. 4: e3001799. 10.1371/journal.pbio.3001799. 37104303 PMC10138789

[ejn70502-bib-0010] Haith, A. M. , D. M. Huberdeau , and J. W. Krakauer . 2015. “Hedging Your Bets: Intermediate Movements as Optimal Behavior in the Context of an Incomplete Decision.” PLoS Computational Biology 11: 1–21. 10.1371/journal.pcbi.1004171 PMC4379031 25821964

[ejn70502-bib-0011] Held, R. , and S. J. Freedman . 1963. “Plasticity in Human Sensorimotor Control.” Science 142: 455–462. 14064442 10.1126/science.142.3591.455

[ejn70502-bib-0012] Herzfeld, D. J. , P. A. Vaswani , M. K. Marko , and R. Shadmehr . 2014. “A Memory of Errors in Sensorimotor Learning.” Science 345: 1349–1353. 25123484 10.1126/science.1253138 PMC4506639

[ejn70502-bib-0002] Houde, J. F. , and M. I. Jordan . 1998. “Sensorimotor Adaptation in Speech Production.” Science 279, no. 5354: 1213–1216. 9469813 10.1126/science.279.5354.1213

[ejn70502-bib-0013] Huang, V. S. , A. Haith , P. Mazzoni , and J. W. Krakauer . 2011. “Rethinking Motor Learning and Savings in Adaptation Paradigms: Model‐Free Memory for Successful Actions Combines With Internal Models.” Neuron 70: 787–801. 21609832 10.1016/j.neuron.2011.04.012 PMC3134523

[ejn70502-bib-0014] Kagerer, F. A. , and J. L. Contreras‐Vidal . 2009. “Adaptation of Sound Localization Induced by Rotated Visual Feedback in Reaching Movements.” Experimental Brain Research 193: 315–321. 19048242 10.1007/s00221-008-1630-3 PMC3203351

[ejn70502-bib-0015] Katz, W. F. , and S. Mehta . 2015. “Visual Feedback of Tongue Movement for Novel Speech Sound Learning.” Frontiers in Human Neuroscience 9: 1–13. 26635571 10.3389/fnhum.2015.00612 PMC4652268

[ejn70502-bib-0016] Kim, H. E. , J. R. Morehead , D. E. Parvin , R. Moazzezi , and R. B. Ivry . 2018. “Invariant Errors Reveal Limitations in Motor Correction Rather Than Constraints on Error Sensitivity.” Communications Biology 1: 19. 30271906 10.1038/s42003-018-0021-y PMC6123629

[ejn70502-bib-0017] Krakauer, J. W. , C. Ghez , and M. F. Ghilardi . 2005. “Adaptation to Visuomotor Transformations: Consolidation, Interference, and Forgetting.” Journal of Neuroscience 25: 473–478. 15647491 10.1523/JNEUROSCI.4218-04.2005 PMC6725486

[ejn70502-bib-0018] Lane, H. , and B. Tranel . 1971. “The Lombard Sign and the Role of Hearing in Speech.” Journal of Speech, Language, and Hearing Research 14: 677–709.

[ejn70502-bib-0019] Mazzoni, P. , and J. W. Krakauer . 2006. “An Implicit Plan Overrides an Explicit Strategy During Visuomotor Adaptation.” Journal of Neuroscience 26: 3642–3645. 16597717 10.1523/JNEUROSCI.5317-05.2006 PMC6674132

[ejn70502-bib-0020] Morehead, J. R. , S. E. E. Qasim , M. J. J. Crossley , and R. B. Ivry . 2015. “Savings Upon Re‐Aiming in Visuomotor Adaptation.” Journal of Neuroscience 35: 14386–14396. 26490874 10.1523/JNEUROSCI.1046-15.2015 PMC4683692

[ejn70502-bib-0021] Morehead, J. R. , J. A. Taylor , D. Parvin , and R. B. Ivry . 2017. “Characteristics of Implicit Sensorimotor Adaptation Revealed by Task‐Irrelevant Clamped Feedback.” Journal of Cognitive Neuroscience 26: 1–14. 10.1162/jocn_a_01108 PMC5505262 28195523

[ejn70502-bib-0022] Opoku‐Baah, C. , A. M. Schoenhaut , S. G. Vassall , D. A. Tovar , R. Ramachandran , and M. T. Wallace . 2021. “Visual Influences on Auditory Behavioral, Neural, and Perceptual Processes: A Review.” Journal of the Association for Research in Otolaryngology 22: 365–386. 34014416 10.1007/s10162-021-00789-0 PMC8329114

[ejn70502-bib-0023] Orban de Xivry, J.‐J. , and P. Lefèvre . 2015. “Formation of Model‐Free Motor Memories During Motor Adaptation Depends on Perturbation Schedule.” Journal of Neurophysiology 113: 2733–2741. 25673736 10.1152/jn.00673.2014 PMC4416610

[ejn70502-bib-0024] Oscari, F. , R. Secoli , F. Avanzini , G. Rosati , and D. J. Reinkensmeyer . 2012. “Substituting Auditory for Visual Feedback to Adapt to Altered Dynamic and Kinematic Environments During Reaching.” Experimental Brain Research 221: 33–41. 22733310 10.1007/s00221-012-3144-2

[ejn70502-bib-0025] Pekny, S. E. , S. E. Criscimagna‐Hemminger , and R. Shadmehr . 2011. “Protection and Expression of Human Motor Memories.” Journal of Neuroscience: The Official Journal of the Society for Neuroscience 31: 13829–13839. 21957245 10.1523/JNEUROSCI.1704-11.2011 PMC3208234

[ejn70502-bib-0026] Pfordresher, P. Q. 2005. “Auditory Feedback in Music Performance: The Role of Melodic Structure and Musical Skill.” Journal of Experimental Psychology. Human Perception and Performance 31: 1331–1345. 16366793 10.1037/0096-1523.31.6.1331

[ejn70502-bib-0027] Pfordresher, P. Q. 2012. “Musical Training and the Role of Auditory Feedback During Performance.” Annals of the New York Academy of Sciences 1252: 171–178. 22524356 10.1111/j.1749-6632.2011.06408.x

[ejn70502-bib-0028] Plack, C. , and A. Oxenham . 2005. “Overview: The Present and Future of Pitch.” Pitch: Neural Coding and Perception. Pitch Neural Coding Percept 24: 1–6.

[ejn70502-bib-0029] Rosati, G. , F. Oscari , S. Spagnol , F. Avanzini , and S. Masiero . 2012. “Effect of Task‐Related Continuous Auditory Feedback During Learning of Tracking Motion Exercises.” Journal of Neuroengineering and Rehabilitation 9: 79. 23046683 10.1186/1743-0003-9-79 PMC3554473

[ejn70502-bib-0030] Sarlegna, F. R. , and R. L. Sainburg . 2009. “The Roles of Vision and Proprioception in the Planning of Reaching Movements.” Advances in Experimental Medicine and Biology 629: 317–335. 19227507 10.1007/978-0-387-77064-2_16 PMC3709263

[ejn70502-bib-0031] Schmitz, G. , and O. Bock . 2014. “A Comparison of Sensorimotor Adaptation in the Visual and in the Auditory Modality.” PLoS ONE 9: e107834. 25254660 10.1371/journal.pone.0107834 PMC4177875

[ejn70502-bib-0032] Shmuelof, L. , V. S. Huang , A. M. Haith , R. J. Delnicki , P. Mazzoni , and J. W. Krakauer . 2012. “Overcoming Motor “Forgetting” Through Reinforcement of Learned Actions.” Journal of Neuroscience 32: 14617–14621. 23077047 10.1523/JNEUROSCI.2184-12.2012 PMC3525880

[ejn70502-bib-0033] Sigrist, R. , G. Rauter , L. Marchal‐Crespo , R. Riener , and P. Wolf . 2014. “Sonification and Haptic Feedback in Addition to Visual Feedback Enhances Complex Motor Task Learning.” Experimental Brain Research 233: 909–925. 25511166 10.1007/s00221-014-4167-7

[ejn70502-bib-0034] Sigrist, R. , G. Rauter , R. Riener , and P. Wolf . 2013. “Augmented Visual, Auditory, Haptic, and Multimodal Feedback in Motor Learning: A Review.” Psychonomic Bulletin & Review 20: 21–53. 23132605 10.3758/s13423-012-0333-8

[ejn70502-bib-0035] Smith, M. A. , A. Ghazizadeh , and R. Shadmehr . 2006. “Interacting Adaptive Processes With Different Timescales Underlie Short‐Term Motor Learning.” PLoS Biology 4: 1035–1043. 10.1371/journal.pbio.0040179 PMC1463025 16700627

[ejn70502-bib-0036] Taylor, J. A. , L. L. Hieber , and R. B. Ivry . 2013. “Feedback‐Dependent Generalization.” Journal of Neurophysiology 109, no. 1: 202–215. 23054603 10.1152/jn.00247.2012 PMC3545161

[ejn70502-bib-0037] Taylor, J. A. , J. W. Krakauer , and R. B. Ivry . 2014. “Explicit and Implicit Contributions to Learning in a Sensorimotor Adaptation Task.” Journal of Neuroscience: The Official Journal of the Society for Neuroscience 34: 3023–3032. 24553942 10.1523/JNEUROSCI.3619-13.2014 PMC3931506

[ejn70502-bib-0038] van Beers, R. J. , D. M. Wolpert , and P. Haggard . 2002. “When Feeling Is More Important Than Seeing in Sensorimotor Adaptation.” Current Biology 12: 834–837. 12015120 10.1016/s0960-9822(02)00836-9

[ejn70502-bib-0039] van Vugt, F. T. , and B. Tillmann . 2015. “Auditory Feedback in Error‐Based Learning of Motor Regularity.” Brain Research 1606: 54–67. 25721795 10.1016/j.brainres.2015.02.026

[ejn70502-bib-0040] Wolpert, D. M. , Z. Ghahramani , and M. I. Jordan . 1995. “Are Arm Trajectories Planned in Kinematic or Dynamic Coordinates? An Adaptation Study.” Experimental Brain Research 103: 460–470. 7789452 10.1007/BF00241505

[ejn70502-bib-0041] Zanotto, D. , G. Rosati , S. Spagnol , P. Stegall , and S. K. Agrawal . 2013. “Effects of Complementary Auditory Feedback in Robot‐Assisted Lower Extremity Motor Adaptation.” IEEE Transactions on Neural Systems and Rehabilitation Engineering 21: 775–786. 23529102 10.1109/TNSRE.2013.2242902

[ejn70502-bib-0042] Zatorre, R. J. , J. L. Chen , and V. B. Penhune . 2007. “When the Brain Plays Music: Auditory‐Motor Interactions in Music Perception and Production.” Nature Reviews. Neuroscience 8: 547–558. 17585307 10.1038/nrn2152

